# First evidence of *Flamingolepis liguloides* in parthenogenetic *Artemia* from a Mediterranean hypersaline lagoon (Salina dei Monaci, Apulia, Italy)

**DOI:** 10.1017/S0031182026102194

**Published:** 2026-06

**Authors:** Marta Favero, Gianpasquale Chiatante, Eleonora Di Basilio, Martina Mazzetti, Giovanni Polverino, Marialetizia Palomba, Daniele Canestrelli

**Affiliations:** Department of Ecological and Biological Sciences, University of Tuscia, Viterbo, Italy

**Keywords:** *Flamingolepis liguloides*, hypersaline habitat, molecular identification, parthenogenetic *Artemia*, Salina dei Monaci

## Abstract

Hypersaline ecosystems host simplified yet highly structured food webs, in which *Artemia* plays a central ecological role, including serving as an intermediate host for several avian cestodes. *Flamingolepis liguloides* is among the most widespread parasites associated with *Artemia* worldwide; however, its presence in Italy has not been confirmed since early reports from Sardinia in the 1990s. Here, we provide the first documented evidence of *F. liguloides* infecting parthenogenetic *Artemia* in the hypersaline lagoon of Salina dei Monaci (Apulia, Italy). Host and parasite identities were confirmed through an integrative approach combining morphological analyses with molecular data, including newly generated 18S and 28S rDNA sequences, the latter providing novel molecular information for the parasite. Among the 254 brine shrimp examined, prevalence reached 93.3%, with a mean abundance of 7.3 ± 10.1 cysticercoids and up to 73 larvae in a single host. Parasite abundance was positively correlated with host body size, consistent with cumulative exposure and size-dependent feeding capacities. Cysticercoids were distributed within the host body, with the thorax harbouring the highest parasite loads, while cysticercoids became progressively more frequent in other body regions in larger individuals. The high infection levels observed, together with the presence of large flamingo aggregations in the lagoon, indicate a stable and active transmission cycle. This study fills an important geographic gap in the known distribution of *F. liguloides* and highlights the Salina dei Monaci as a promising natural system for future investigations on host–parasite dynamics in Mediterranean hypersaline environments.

## Graphical abstract

**Figure:**
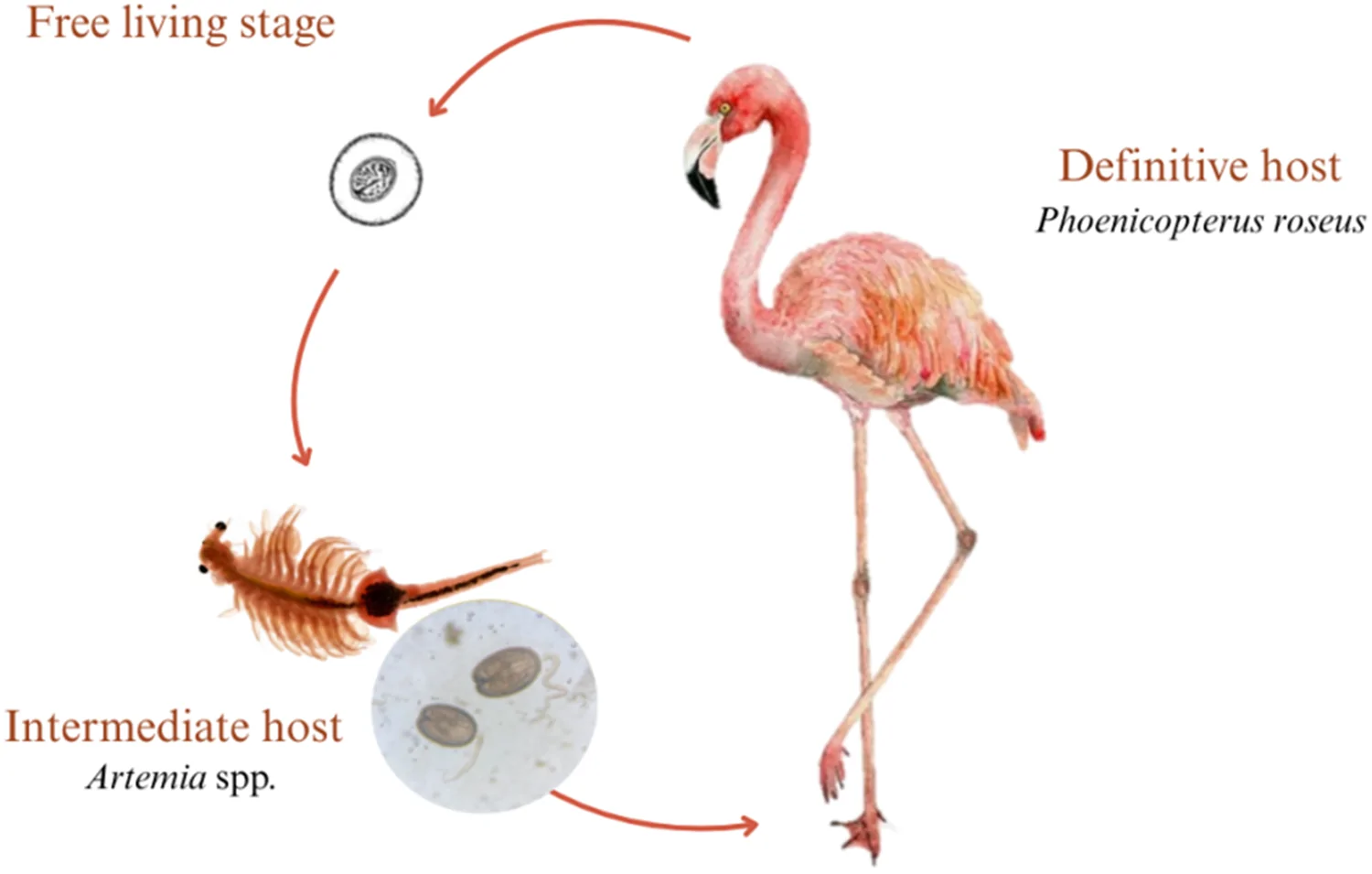

## Introduction

Hypersaline environments represent unique ecological systems shaped by extreme abiotic conditions, where only highly specialized and uniquely adapted organisms can survive (Colletti, 2014). Despite their apparent simple and reduced biodiversity, these ecosystems play a crucial role in local ecological balance, hosting complex food webs in which a limited number of taxa support multiple trophic interactions (Saccò et al., 2021). Among the key components of these systems are brine shrimp of the genus *Artemia* (Leach, 1819; Branchiopoda: Anostraca), which act as primary consumers and constitute an essential link between primary producers and higher trophic levels (Gajardo and Beardmore, 2012). Brine shrimp are filter-feeders primarily consuming microalgae and planktonic bacteria, thus regulating their populations and contributing to nutrient cycling (Belovsky et al., 2011; Sánchez et al., 2016). At the same time, they serve as important food resources for several waterbird species (Sánchez et al., 2006; Dolmans et al., 2025), thereby establishing intricate ecological interactions, including predator–prey dynamics and multiple host–parasite relationships (Sánchez et al., 2013). Notably, brine shrimp are recognized as intermediate hosts for numerous avian cyclophyllidean cestodes (Georgiev et al., 2005, 2007; Amarouayache et al., 2009; Sánchez et al., 2013; Redón et al., 2015; Sivakumar et al., 2020). Although various studies on cestode–*Artemia* interactions originate from the American continent (Young, 1952; Redón et al., 2015, 2019; Guagliardo et al., 2020), most of them regard the Mediterranean area where the cestode *Flamingolepis liguloides* (Gervais, 1847) is particularly relevant in *Artemia* populations (Amat et al., 1991a; Georgiev et al., 2007, 2014; Redón et al., 2011; Sánchez et al., 2013). The parasite’s life cycle is closely intertwined with the ecology of brine shrimp and flamingos (Phoenicopteridae), which are among their avian predators. *Artemia* acquires infection while filter-feeding, ingesting cestode eggs containing an oncosphere larva (Robert and Gabrion, 1991). The oncosphere penetrates the intestinal wall and develops into a cysticercoid in the hemocoel (Sánchez et al., 2007). Transmission to the definitive host occurs when infected brine shrimp are consumed by flamingos, where the parasite reaches maturity and releases eggs into the environment. *Flamingolepis liguloides* has been reported from multiple *Artemia* populations across the Mediterranean region – such as France (Robert and Gabrion, 1991) and Spain (Amat et al., 1991a; Georgiev et al., 2007) – as well as from more distant regions, including Tengiz Lake in Kazakhstan (Maksimova, 1973) and the Godolphin Lakes in Dubai (Sivakumar et al., 2020). In Italy, evidence for the presence of this cestode is scarce, with the only published records dating back to the early 1990s in southwestern Sardinia (Di Cave et al., 1990; Di Cave and Mura, 1990; Mura, 1995). To date, no additional occurrences have been reported elsewhere in the country. To fill this gap, the present study investigates the presence of *F. liguloides* in *Artemia* sp. within the hypersaline lagoon of ‘Saline dei Monaci’ (Apulia Region, Italy). The aim is to provide the first evidence of this parasite in the local *Artemia* population and to contribute new information on host–parasite interactions in Mediterranean hypersaline ecosystems.

## Materials and methods

### Study area

The study was conducted in the Salina dei Monaci (40°18′7.281″ N, 17°43′54.768″ E), a coastal hypersaline lagoon located in Manduria (Taranto, Apulia Region, Southern Italy). Formerly a saltwork, it is a shallow basin (20–70 cm) that depends mainly on rainfall for its filling, after which evaporation leads to progressive concentration and the creation of hypersaline conditions. Salinity and temperature vary seasonally, ranging from 40 gL^−1^ in winter to 290 gL^−1^ in summer and from 5–6 °C in winter to 37 °C in summer, respectively (Mura et al., 2005). Following the cessation of salt extraction in the mid-20th century, the area has developed into an ecologically valuable wetland supporting numerous resident and migratory waterbirds and is currently designated as a Special Area of Conservation (SAC IT IT9130001 ‘Torre Colimena’) due to its botanical and ecological relevance (Mastrolia et al., 2017). Together with the adjacent Dunes of Torre Colimena, the area is protected by ‘Riserve Naturali Regionali Orientate del Litorale Tarantino Orientale’.

### Sample collection and parasitological analyses

A random sample of *Artemia* sp. individuals was collected in October 2023 from the Salina dei Monaci, using a plankton hand net with a 125 μm mesh size. Live specimens were transported to the CISMAR laboratories (Ichthyogenic Experimental Marine Centre, Tuscia University) in 30 L aerated plastic containers to ensure survival during transport. Within 24 h of sampling, 254 randomly selected *Artemia* individuals were identified to species level using morphological keys (Mura and Brecciaroli, 2004), measured for total length to the nearest 0.1 mm under a stereomicroscope (Leica M205 FCA) and examined for parasitic infections. Cysticercoids were isolated and counted in each infected host and identified to species level based on diagnostic morphological characters described by Georgiev et al. (2005).

### Molecular identification of host and parasite

To confirm the species identity of both host and parasite, total genomic DNA was extracted from 15 brine shrimp individuals and 10 *F. liguloides* cysticercoids randomly selected from the overall sample using the Quick-gDNA Miniprep Kit (Zymo Research), following the manufacturer’s instructions. A partial fragment of the mitochondrial cytochrome c oxidase subunit I (*cox*1) gene was amplified from each brine shrimp specimen using the primers 1/2COI_Fol-F (5′-ATT CTA CGA ATC ACA AGG ATA TTG G-3′) and 1/2COI_Fol-R (5′-TAC ACT TCA GGA TGG CCA AAA AAT CA-3′) (Muñoz et al., 2008). Polymerase chain reactions (PCRs) were carried out in a 25 μL volume containing 0.6 μL of each primer 10 μM (Promega), 2 μL of MgCl_2_ 25 mM (Promega), 5 μL of 5× buffer (Promega), 0.6 μL of dNTPs 10 mM (Promega), 0.2 μL of Go-Taq Polymerase (5 U μL^−1^) (Promega) and 2 μL of total DNA. PCR temperature conditions were as follows: an initial denaturation at 94 °C for 3 min, followed by 40 cycles of denaturation at 94 °C for 45 sec, annealing at 55 °C for 1 min and extension at 72 °C for 1 min, with a final extension at 72 °C for 5 min.

For each *F. liguloides* cysticercoid, a partial fragment of the small ribosomal subunit gene (SSU, 18S) was amplified using the primers HymF1 (5′-GAT CAA TTG GAG GGC AAG T-3′) and HymR1 (5′-CTG TCA TGA CGG TGA TTG A-3′) (Redón et al., 2024). The partial large ribosomal subunit (LSU, 28S) was also amplified for the first time using the primers ZX-1 (5′-ACCCGCTGAATTTAAGCATAT-3′) and 1500R (5′-GCT ATC CTG AGG GAA ACT TCG-3′) (Van der Auwera et al., 1994; Palm et al., 2009). Both PCRs were carried out in a 25 μL volume containing 0.6 μL of each primer 10 μM (Promega), 2 μL of MgCl_2_ 25 mM (Promega), 5 μL of 5× buffer (Promega), 0.6 μL of dNTPs 10 mM (Promega), 0.2 μL of Go-Taq Polymerase (5 U μL^−1^) (Promega) and 2 μL of total DNA. PCR temperature conditions were as follows: an initial denaturation at 95 °C for 5 min, followed by 40 cycles of denaturation at 95 °C for 30 sec, annealing at 55 °C (28S) and 54 °C (18S) for 30 sec and extension at 72 °C for 2 min, with a final extension at 72 °C for 7 min. The successful PCR products were purified, and Sanger sequenced through an Automated Capillary Electrophoresis Sequencer 3730 DNA Analyzer (Applied Biosystems), using the Big-Dye® Terminator v3.1 Cycle Sequencing Kit (Life Technologies). Forward and reverse sequences obtained were assembled using Sequencher v. 5.0.1 (GeneCodes Co.) and compared with reference sequences using BLASTn (Morgulis et al., 2008).

### Statistical analyses

Prevalence (P: proportion of infected individuals), mean abundance (MA: mean number of cysticercoids per host examined) ± standard deviation (± SD) and mean intensity (MI: mean number of cysticercoids in infected hosts) ± SD were calculated following Bush et al. (1997) on the total sample of individuals examined (*N* = 254).

The relationship between brine shrimp total length and parasite abundance was assessed using linear regression. Based on a preliminary graphical analysis, we fitted 2 models: one with parasite abundance as a linear term (*y* ∼ *x*) and one including a quadratic term (*y* ∼ *x* + *x*^2^). These models were compared using the second-order Akaike’s information criterion (AICc; Burnham and Anderson, 2002). The model with the lowest AICc was considered the best, provided that its AICc differed from competing models by at least 2 units (Burnham and Anderson, 2002).

In addition, the relationship between the number of cysticercoids in each body part (head, thorax, abdomen, ovisac) and the total parasite abundance was assessed using a generalized linear model with a negative binomial error distribution (Zuur et al., 2007; Lindén and Mäntyniemi, 2011). Specifically, we modelled the number of cysticercoids per body part as the response variable, including the total parasite abundance, the body length and interactions between total parasite abundance × body part and body length × body part as predictors. Model support was evaluated by comparing the AICc of this model with that of a null model (i.e. without predictors, *y* ∼ 1; Burnham and Anderson, 2002). Multicollinearity was assessed using variance inflation factors (VIFs), model fit was quantified using pseudo *R*^2^ (Nakagawa and Schielzeth, 2013) and the normality of residuals was checked with a Kolmogorov–Smirnov test. All statistical analyses were performed in R version 4.2.2 (R Core Team, 2022) and the packages *MuMIn* (Bartoń, 2018), *glmmTMB* (Brooks et al., 2017) and *DHARMa* (Hartig, 2022).

## Results

### Identification of parthenogenetic *Artemia* and *F. liguloides*

**Figure 1.**
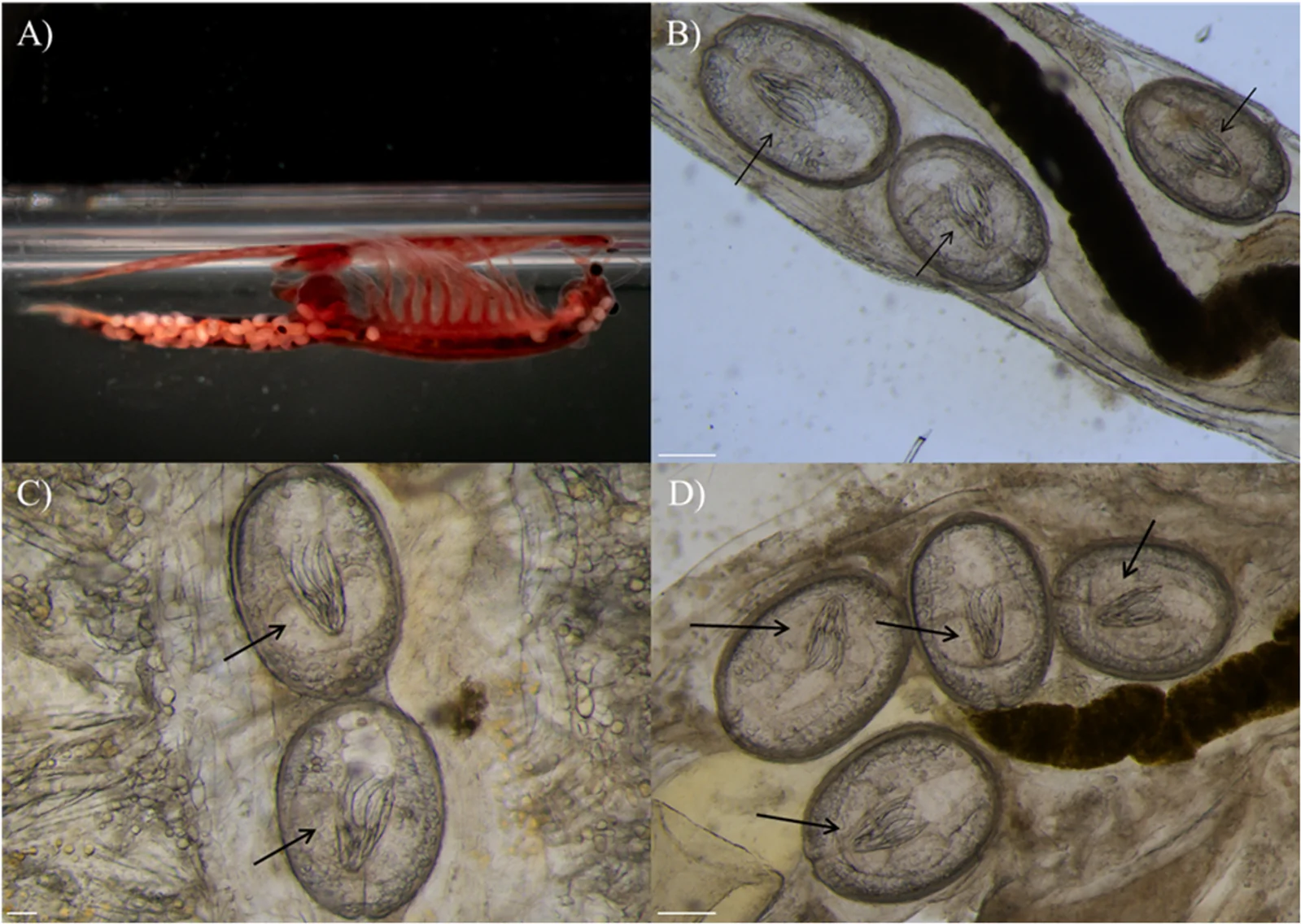
Spatial distribution of *Flamingolepis liguloides* cysticercoids in parthenogenetic *Artemia*. (A) General view illustrating cysticercoids distributed throughout the whole body, captured with a Nikon D3500 camera, equipped with a 55 mm 3.5 Micro-Nikkor Macro lens. (B–D) Examples of cysticercoids in specific anatomical regions with the rostellar apparatus visible and indicated by black arrows, captured using a Leica M205 FCA stereomicroscope: (B) abdomen (white scale bar = 250 µm); (C) thorax (white scale bar = 100 µm) and (D) near the ovisac (white scale bar = 250 µm). Figure 1 long description.

Morphological analyses of the brine shrimp collected from the Salina dei Monaci confirmed their assignment to the genus *Artemia* based on diagnostic characters, including the typical anostracan body plan, 11 pairs of thoracopods and the absence of males, all consistent with parthenogenetic populations (Browne et al., 1991; Figure 1A). Partial sequences of the mitochondrial *cox1* gene were successfully obtained from all 15 *Artemia* individuals analysed (fragment length: 600 bp). BLAST comparisons revealed that all sequences shared 100% identity with reference sequences of *Artemia* parthenogenetic lineage deposited in GenBank (MT791655; Rode et al., 2022), confirming their molecular identification.

Morphological examination of cestode specimens identified diagnostic features consistent with *F. liguloides* according to Georgiev et al. (2005), including a rostellar apparatus with 8 slender hooks (∼130 μm total length; Figure 1B–D). Partial sequences of the ribosomal 18S and 28S genes were successfully obtained from all 10 *F. liguloides* individuals analysed (fragment length: 1100 bp and 1516 bp, respectively). BLAST comparisons revealed that all 18S sequences shared 100% identity with reference sequences of *F. liguloides* deposited in GenBank (OR995639; Redón et al., 2024). BLAST comparisons of 28S sequences shared 88.6% identity with the cestode *Hymenolepis microstoma* deposited in GenBank (LC064144; Nkouawa et al., 2016). The newly obtained sequences for parthenogenetic *Artemia* and *F. liguloides* have been deposited in GenBank under accession numbers PZ315227 (*cox*1) and PZ315369–PZ315425 (18S–28S, respectively).

### Parasitic infection and site preferences

**Figure 2.**
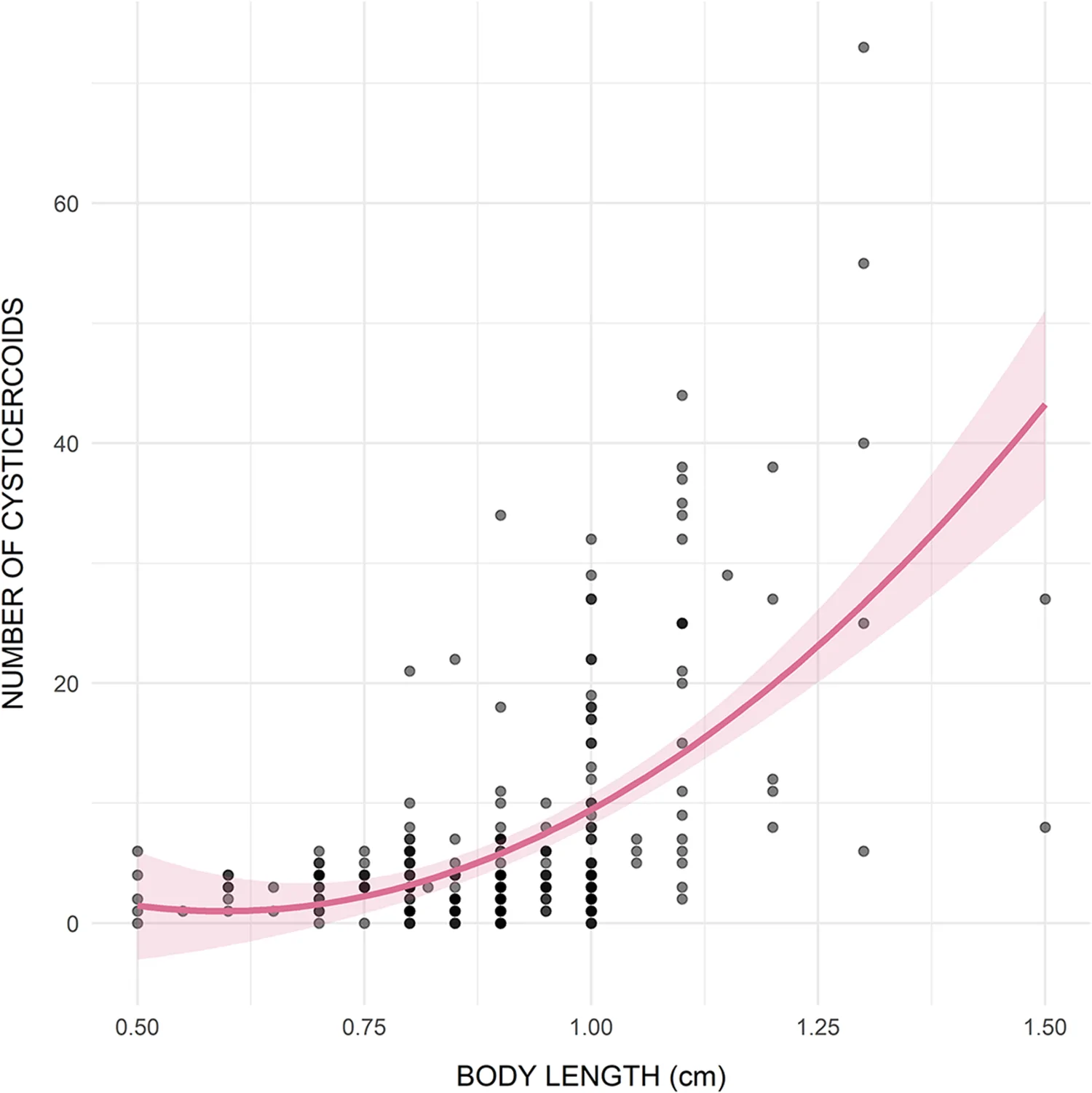
Relationship between host body length (cm) and cysticercoid abundance. Figure 2 long description.

Infection with *F. liguloides* was detected in 93.3% of the examined brine shrimp individuals. Mean abundance was 7.3 ± 10.1 cysticercoids per host, while mean intensity reached 7.8 ± 10.3 cysticercoids per infected host (range: 1–73), with a maximum of 73 cysticercoids in a single individual. Parasite abundance significantly increased with host body length, with the best-fitting model including a positive quadratic term (Table 1; *R*^2^ = 0.343) (Figure 2).

**Table 1.** Comparison of models investigating the relationship between the total number of cysticercoids of *F. liguloides* and the body size of parthenogenetic *Artemia*. Comparison between linear and quadratic models showed that the latter provided the best fit Table 1 long description.

Model			logLik	AICc	ΔAICc	Σ*w_i_*
Parasite abundance ∼ body length + body length^2^			−889.447	1787.1	0.00	1.00
Parasite abundance ∼ body length			−899.623	1805.3	18.29	0.00

Variable	Estimate	SE	95% LCI	95% UCI	*p*	
Intercept	19.249	9.88	–	–	–	
Body length	−61.34	21.15	−103.00	−19.68	0.004	
Body length^2^	51.55	11.27	29.36	73.74	<0.001	

Reported are the log-likelihood (logLik), second-order Akaike’s information criterion (AICc), differences in AICc (ΔAICc) and Akaike weights (Σ*w_i_*). Summary statistics of the best-supported model include parameter estimates, standard errors (SEs), 95% confidence intervals (LCI: lower, UCI: upper) and their significance (*P*).

**Figure 3.**
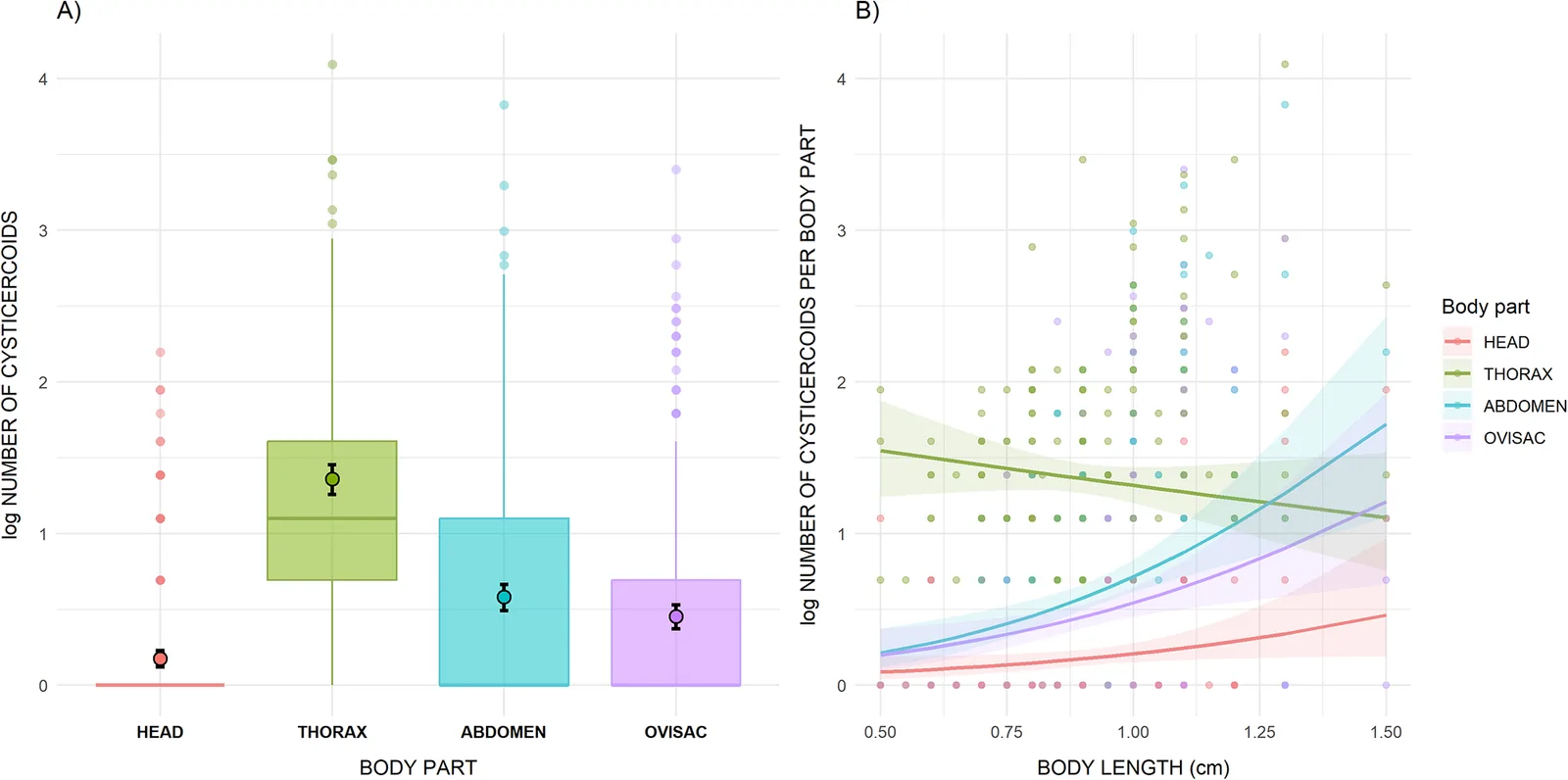
(A) Abundance of *F. liguloides* cysticercoids in each body part (head, thorax, abdomen, ovisac) of parthenogenetic *Artemia*. Filled dots represent values predicted by the model. (B) Relationship between cysticercoid abundance per body part, total cysticercoid number per individual and the body length of parthenogenetic *Artemia*. Lines represent values predicted by the model, whereas the shaded areas indicate 95% confidence intervals. In both panels, the *y*-axis is log-transformed to improve visualization. Figure 3 long description.

Cysticercoids were distributed within the host body, occurring predominantly in the thorax (55.2%), followed by the abdomen (22.6%), the ovisac region (17.0%) and the head (5.3%). According to the negative binomial model (Table 2), the thorax harboured significantly (*p* < 0.001) more cysticercoids than the head, abdomen or ovisac (Figure 3A). However, the significant interaction between body length and body part indicates that in larger brine shrimp, which hosted a higher cysticercoid abundance, the relative number of cysticercoids in the thorax decreases compared to the other body parts (Figure 3B). The model showed no collinearity (VIF < 3), had a good level of explained variance (*R*^2^ = 0.604) and exhibited normally distributed residuals (Kolmogorov–Smirnov test, *p* = 0.544). None of the infected brine shrimp, at any level of infection, contained cysts or nauplii in the ovisac, whereas cysts were observed exclusively in uninfected individuals. Because the number of cyst-bearing individuals was very low, statistical significance could not be assessed.

**Table 2.** Model comparison assessing the effects of body part (head, thorax, abdomen, ovisac), total cysticercoid abundance, body length of parthenogenetic *Artemia* and their interactions on the number of *F. liguloides* cysticercoids per body part Table 2 long description.

Model		logLik	AICc	ΔAICc	Σ*w_i_*
Parasite abundance per body part ∼ parasite abundance + body length + parasite abundance × body part + body length × body part		−1315.88	2658.1	0.00	1.00
Parasite abundance per body part ∼ 1		−1659.28	3324.6	666.47	0.00

Variable	Estimate	SE	95% LCI	95% UCI	*P*
Intercept	−3.893	0.821	–	–	–
Parasite abundance	0.075	0.012	0.051	0.099	<0.001
Body length	1.881	0.893	0.130	3.632	0.035
Body part | thorax	4.953	0.921	3.148	6.758	<0.001
Body part | abdomen	0.391	1.047	−1.662	2.444	0.709
Body part | ovisac	0.535	1.098	−1.618	2.688	0.626
Parasite abundance × body part | thorax	0.001	0.015	−0.028	0.030	0.933
Parasite abundance × body part | abdomen	0.007	0.016	−0.024	0.038	0.658
Parasite abundance × body part | ovisac	0.016	0.016	−0.015	0.047	0.309
Body length × body part | thorax	−2.483	1.017	−4.477	−0.488	0.015
Body length × body part | thorax	1.078	1.148	−1.173	3.329	0.348
Body length × body part | thorax	0.493	1.203	−1.865	2.851	0.682

Comparison with the null model indicated that several predictors contributed to explaining variation in parasite counts. Reported are the log-likelihood (logLik), second-order Akaike’s information criterion (AICc), differences in AICc (ΔAICc) and Akaike weights (Σ*w_i_*). For the best-supported model, we present parameter estimates, standard errors (SEs), 95% confidence intervals (LCI: lower; UCI: upper) and significance levels (*p*). The head is the reference level for the body part factor.

## Discussion

This study provides the first confirmed evidence, supported by morphological and molecular data, of a host–parasite association between parthenogenetic *Artemia* populations – considered a complex of genetically heterogeneous, often polyploid lineages (Asem et al., 2024 and references therein) – and *F. liguloides* in the hypersaline lagoon of Salina dei Monaci (Apulia, Italy), with newly generated 28S rDNA sequences obtained for the parasite. Previous investigations in the Salina dei Monaci area reported the presence of parthenogenetic *Artemia* but did not document any associated parasite fauna (Moscatello et al., 2002; Moscatello and Belmonte, 2004; Mura et al., 2005). However, our findings represent the first verified occurrence of *F. liguloides* in Italy since the early reports from southwestern Sardinia in the 1990s, where the parasite was recorded in bisexual *Artemia* populations from Su Pallosu pond with low prevalence and abundance (approximately 2–3%; Di Cave et al., 1990; Di Cave and Mura, 1990; Mura, 1995).

In contrast to those earlier observations, the population examined in the present study exhibited high infection levels, with a prevalence of approximately 90% and a significant increase in parasite burden with host body size. Such high infection levels strongly suggest elevated environmental parasite pressure and indicate that the life cycle of *F. liguloides* is fully maintained within the Salina dei Monaci ecosystem. The coexistence of dense parthenogenetic *Artemia* populations with large aggregations of the definitive host, the greater flamingo, likely plays a key role in sustaining parasite transmission (Sánchez et al., 2013). During the sampling period (October), approximately 200 flamingos were observed in the lagoon (GC, pers. obs.), consistent with the species’ main seasonal presence and aggregation in Mediterranean wetlands during post-breeding and post-fledging movements (Johnson, 1989; Spina and Volponi, 2008). Indeed, as flamingo faeces represent the primary source of infective cestode eggs, persistent bird aggregations are expected to substantially increase environmental infection pressure on *Artemia* populations. This scenario likely did not apply to the studies conducted in the early 1990s (Di Cave et al., 1990; Di Cave and Mura, 1990; Mura, 1995). Flamingos were absent from Su Pallosu pond in 1991 (Mura, 1995), sampling was carried out between January and March (i.e. outside the main period of flamingo movements) and the species was still relatively uncommon in the Mediterranean at that time, with no breeding colonies in Italy (the first breeding event in Sardinia was recorded in 1993; Grussu, 1993). Consequently, the probability of flamingo movements, and thus parasite dispersal, across Mediterranean wetlands was likely low.

The high infection levels observed in this population revealed a positive relationship between host body size and parasite burden, in line with patterns described for trophically transmitted endoparasites and supporting the hypothesis of parasite accumulation over time (e.g. Cipriani et al., 2018; Gay et al., 2018; Levsen et al., 2022; Palomba et al., 2023). In *Artemia*, which has a relatively short life cycle (approximately 30–40 days; Browne et al., 1984) but feeds continuously by filtration, larger individuals are expected to experience longer exposure times and higher probabilities of ingesting infective stages. Previous studies have shown that susceptibility to infection in *Artemia* is developmentally constrained by particle ingestion capacity. Early larval stages (stages III and IV, <3 mm) are able to ingest only particles up to 25–30 μm in diameter (Dobbeleir et al., 1980; Mura, 1995; Fernández, 2001), a size range that excludes *F. liguloides* eggs (typically 40–50 μm). Accordingly, infection is expected to occur only after individuals reach a minimum body size (minimum reported size of infected individuals: 3–4 mm). In the present study, parasitized hosts had a minimum body length of 5 mm, and parasite burden increased progressively with host size above this threshold. Although smaller individuals were not sampled, precluding direct inference on infection at earlier stages, the observed size-dependent increase in parasite load is consistent with cumulative exposure.

As parasite loads increased, spatial constraints within the host appeared to influence cysticercoid distribution. Cysticercoids were predominantly located in the thoracic region, which harboured approximately 55% of the total parasite load, with a maximum of 73 cysticercoids recorded per host, in agreement with previous studies on *F. liguloides* in *Artemia* spp. (Mura, 1995; Koru, 2022). With increasing host size and parasite burden, cysticercoids were increasingly detected in other body regions, including the abdomen, ovisac and head. This progressive expansion across body regions suggests that the thorax represents the primary site of infection, whereas other regions act as secondary sites once the thoracic cavity becomes saturated, as previously proposed for this host–parasite system (Amarouayache et al., 2009). Beyond spatial effects, infections were also associated with marked impacts on host reproduction. All parasitized adult exhibited empty ovarian sacs, indicating functional castration. Parasite-induced castration has been repeatedly documented in *Artemia* infected by cestode cysticercoids (Amat et al., 1991a,b; Sánchez et al., 2012) and is widely interpreted as an adaptive strategy that enhances parasite fitness. By diverting host resources from reproduction to somatic growth and maintenance, castration may increase host longevity and body size, thereby improving parasite survival and increasing the likelihood of trophic transmission to definitive hosts (Amat et al., 1991b).

Taken together, these results indicate the presence of a stable trophic pathway linking intermediate and definitive hosts in the Salina dei Monaci lagoon. Parasites with complex life cycles depend on intact trophic interactions and stable host communities, and disruptions caused by pollution or habitat degradation may interrupt parasite transmission (Marcogliese, 2004; Sures, 2004; Hudson et al., 2006; Palm and Rückert, 2009). Conversely, high parasite burdens generally occur only in systems where food webs remain structurally intact. In this context, the infection patterns of *F. liguloides* reinforce the view that trophically transmitted helminths, when characterized by well-resolved life cycles, host specificity and stable occurrence, can act as sensitive bioindicators of ecosystem health and ecological integrity, rather than merely as stressors (Sures, 2004, 2008; Hudson et al., 2006; Palm and Rückert, 2009; Palomba et al., 2023).

## Figure 1 Long description

The image A shows a specimen in a tube. The image B shows a microscopic view with arrows pointing to oval shapes. The image C shows a microscopic view with arrows pointing to oval shapes. The image D shows a microscopic view with arrows pointing to oval shapes.

Navigate back to Figure 1.

## Figure 2 Long description

The horizontal axis represents BODY LENGTH (cm), ranging from 0.50 to 1.50. The vertical axis represents NUMBER OF CYSTICERCOIDS, ranging from 0 to 60. The plot shows a positive correlation, where the number of cysticercoids generally increases with body length. A curved line indicates the trend, with a shaded band representing the confidence interval. Data points are densely clustered around body lengths of 0.75 to 1.00 cm with lower cysticercoid counts, while higher counts are observed at greater body lengths. Notable outliers appear at body lengths above 1.25 cm with cysticercoid counts exceeding 50. The plot aims to demonstrate that larger body lengths are associated with higher cysticercoid abundance.

Navigate back to Figure 2.

## Figure 3 Long description

The image A showing a box plot labeled A). The horizontal axis is labeled BODY PART with categories HEAD, THORAX, ABDOMEN and OVISAC. The vertical axis is labeled LOG NUMBER OF CYSTICERCOIDS with tick labels 0, 1, 2, 3 and 4. The THORAX box is positioned higher than the other body parts, with its median above 1. The HEAD box is at 0 with points clustered at 0 and a small number of points extending upward to around 2. The ABDOMEN box has a median below 1 with points extending upward to around 4. The OVISAC box has a median below 1 with points extending upward to around 3. The image B showing a scatter plot labeled B). The horizontal axis is labeled BODY LENGTH (mm) with tick labels 0.50, 0.75, 1.00, 1.25 and 1.50. The vertical axis is labeled LOG NUMBER OF CYSTICERCOIDS PER BODY PART with tick labels 0, 1, 2, 3 and 4. A legend titled Body part lists HEAD, THORAX, ABDOMEN and OVISAC, with separate point and line styles for each body part. Multiple points are plotted for each body part across the body-length range. Four smooth lines are shown with shaded bands around them. The THORAX line slopes downward from higher values at shorter body length toward lower values at longer body length. The ABDOMEN line slopes upward across the body-length range. The OVISAC line slopes upward across the body-length range. The HEAD line stays near 0 and rises slightly at longer body length. Across both images, A shows the distribution of log number of cysticercoids by BODY PART and B shows how LOG NUMBER OF CYSTICERCOIDS PER BODY PART varies with BODY LENGTH (mm) for the same body-part categories.

Navigate back to Figure 3.

## Table 1 Long description

The table compares two models linking parasite abundance to host body length: a linear model and a model that also includes body length squared. Model fit metrics show the body length plus squared term model is strongly supported, with log-likelihood minus 889.447, AICc 1787.1, delta AICc 0.00, and Akaike weight 1.00. The linear-only model fits much worse, with log-likelihood minus 899.623, AICc 1805.3, delta AICc 18.29, and weight 0.00. For the best-supported model, the intercept estimate is 19.249 with standard error 9.88, and confidence limits and p-value are not reported. The body length term is negative (estimate minus 61.34, standard error 21.15) with a 95% confidence interval from minus 103.00 to minus 19.68 and p-value 0.004. The body length squared term is positive (estimate 51.55, standard error 11.27) with a 95% confidence interval from 29.36 to 73.74 and p-value less than 0.001. Together, the negative length term and positive squared term indicate a curved relationship rather than a straight-line trend.

Navigate back to Table 1.

## Table 2 Long description

The table compares two statistical models predicting the number of cysticercoids per body part using body part, total cysticercoid abundance, body length, and their interactions. The full model had log-likelihood minus 1315.88 and AICc 2658.1, while the null model had log-likelihood minus 1659.28 and AICc 3324.6, a very large AICc difference of 666.47; the full model carried essentially all model weight. In the best model, total abundance was positively associated with per-part counts (estimate 0.075, confidence interval 0.051 to 0.099, p less than 0.001). Body length also showed a positive main effect (estimate 1.881, confidence interval 0.130 to 3.632, p 0.035). Relative to the head reference, the thorax had much higher counts (estimate 4.953, confidence interval 3.148 to 6.758, p less than 0.001), while abdomen and ovisac differences were not statistically supported (p 0.709 and 0.626). Interactions between total abundance and body part were small and not significant for thorax, abdomen, or ovisac (all p values above 0.30). The body length interaction for the thorax was negative and significant (estimate minus 2.483, confidence interval minus 4.477 to minus 0.488, p 0.015), indicating the length effect differs in the thorax compared with the head. The final two body-length-by-body-part rows appear to be mislabeled as thorax; interpret those specific labels cautiously.

Navigate back to Table 2.

## References

[ref1] Amarouayache M, Derbal F and Kara MH (2009) The parasitism of *Flamingolepis liguloides* (Gervais, 1847) (Cestoda, Hymenolepididae) in *Artemia salina* (Crustacea, Branchiopoda) in two saline lakes in Algeria. *Acta Parasitologica* 54, 330–334. 10.2478/s11686-009-0049-8.

[ref2] Amat F, Gozalbo A, Navarro JC, Hontoria F and Varó I (1991b) Some aspects of *Artemia* biology affected by cestode parasitism. *Hydrobiologia* 212, 39–44. 10.1007/BF00025985.

[ref3] Amat F, Illescas MP and Fernandez J (1991a) Brine shrimp *Artemia* parasitized by *Flamingolepis liguloides* (Cestoda, Hymenolepididae) cysticercoids in Spanish Mediterranean salterns Quantitative aspects. *Vie et Milieu / Life & Environment* 41, 237–244.

[ref4] Asem A, Gajardo G, Hontoria F, Yang C, Shen C-Y, Rastegar-Pouyani N, Padhye SM and Sorgeloos P (2024) The species problem in Artemia Leach, 1819 (Crustacea: Anostraca), a genus with sexual species and obligate parthenogenetic lineages. *Zoological Journal of the Linnean Society* 202, zlad192. 10.1093/zoolinnean/zlad192.

[ref5] Bartoń K (2018) *Package MuMIn: Multi-Model Inference*. Wien.

[ref6] Belovsky GE, Stephens D, Perschon C, Birdsey P, Paul D, Naftz D, Baskin R, Larson C, Mellison C, Luft J, Mosley R, Mahon H, Van Leeuwen J and Allen DV (2011) The Great Salt Lake Ecosystem (Utah, USA): Long term data and a structural equation approach. *Ecosphere* 2, art33. 10.1890/ES10-00091.1.

[ref7] Brooks ME, Kristensen K, van Benthem KJ, Magnusson A, Berg CW, Nielsen A, Skaug HJ, Machler M and Bolker BM (2017) glmmTMB balances speed and flexibility among packages for zero-inflated generalized linear mixed modeling. *The R Journal* 9, 378–400. 10.32614/RJ-2017-066.

[ref8] Browne RA, Sallee SE, Grosch DS, Segreti WO and Purser SM (1984) Partitioning genetic and environmental components of reproduction and lifespan in *Artemia*. *Ecology* 65, 949–960. 10.2307/1938067.

[ref9] Browne RA, Sorgeloos P and Trotman CNA (eds) (1991) *Artemia Biology*. Coca Raton, FL: CRC Press.

[ref10] Burnham KP and Anderson DR (eds) (2002) *Model Selection and Multimodel Inference*. New York, NY: Springer. 10.1007/b97636.

[ref11] Bush AO, Lafferty KD, Lotz JM and Shostak AW (1997) Parasitology meets Ecology on its own terms: Margolis et al. revisited. *The Journal of Parasitology* 83, 575–583. 10.2307/3284227. 9267395

[ref12] Cipriani P, Sbaraglia GL, Paoletti M, Giulietti L, Bellisario B, Palomba M, Bušelić I, Mladineo I, Nascetti G and Mattiucci S (2018) The Mediterranean European hake, *Merluccius merluccius*: Detecting drivers influencing the *Anisakis* spp. larvae distribution. *Fisheries Research* 202, 79–89. 10.1016/j.fishres.2017.07.010.

[ref13] Colletti L (ed) (2014) *La Riserva Naturale Statale “Saline di Tarquinia”* Rome: Corpo forestale dello Stato, Ufficio territoriale per la Biodiversità.

[ref14] Di Cave D, Cirillo L, Dettori C and Mura G (1990) Artemia salina e Branchinella spinosa (Crustacea, Anostraca) ospiti intermedi di Flamingolepis liguloides.p. Cagliari.

[ref15] Di Cave D and Mura G (1990) *Flamingolepis liguloides parasite sur Artemia salina et Branchinella spinosa des milieux mesohalines et hyperalines de Sardaigne (Italie).* Septieme Congres International de Parasitologie. Paris: BC Diffusion 288.

[ref16] Dobbeleir J, Adam N, Bossuyt E, Bruggeman E and Sorgeloos P (1980) New aspects of the use of inert diets for high density culturing of brine shrimp. In Persoone G, Sorgeloos P, Roels O and Jaspers E (eds.), *The Brine Shrimp Artemia. Ecology, Culturing, Use in Aquaculture*. Wetteren Belgium: Universa Press, pp. 165–174.

[ref17] Dolmans V, Partoens L, Deboelpaep E, Horváth Z and Vanschoenwinkel B (2025) Dietary data suggest large branchiopods may be an underrecognised food resource for a wide range of waterbirds. *Hydrobiologia* 852, 4297–4313. 10.1007/s10750-025-05810-8.

[ref18] Fernández RG (2001) Artemia bioencapsulation I. Effect of particle sizes on the filtering behavior of *Artemia franciscana*. *Journal of Crustacean Biology* 21, 435–442. 10.1163/20021975-99990144.

[ref19] Gajardo GM and Beardmore JA (2012) The brine shrimp *Artemia*: Adapted to critical life conditions. *Frontiers in Physiology* 3. 10.3389/fphys.2012.00185. PMC3381296 22737126

[ref20] Gay M, Bao M, MacKenzie K, Pascual S, Buchmann K, Bourgau O, Couvreur C, Mattiucci S, Paoletti M, Hastie LC, Levsen A and Pierce GJ (2018) Infection levels and species diversity of ascaridoid nematodes in Atlantic cod, *Gadus morhua*, are correlated with geographic area and fish size. *Fisheries Research* 202, 90–102. 10.1016/j.fishres.2017.06.006.

[ref21] Georgiev B, Sánchez M, Green A, Nikolov P, Vasileva G and Mavrodieva R (2005) Cestodes from *Artemia parthenogenetica* (Crustacea, Branchiopoda) in the Odiel Marshes, Spain: A systematic survey of cysticercoids. *Acta Parasitologica* 50, 105–117.

[ref22] Georgiev BB, Angelov A, Vasileva GP, Sánchez MI, Hortas F, Mutafchiev Y, Pankov P and Green AJ (2014) Larval helminths in the invasive American brine shrimp *Artemia franciscana* throughout its annual cycle. *Acta Parasitologica* 59, 380–389. 10.2478/s11686-014-0255-x. 25119350

[ref23] Georgiev BB, Sánchez MI, Vasileva GP, Nikolov PN and Green AJ (2007) Cestode parasitism in invasive and native brine shrimps (*Artemia* spp.) as a possible factor promoting the rapid invasion of *A. franciscana* in the Mediterranean region. *Parasitology Research* 101, 1647–1655. 10.1007/s00436-007-0708-3. 17712569

[ref24] Grussu M (1993) Buone notizie dalla Sardegna (nidificazione di *Phoenicopterus ruber* e di *Ciconia ciconia*). *BW Rivista Italiana Di Birdwatching* 1(3), 65–66.

[ref25] Guagliardo S, Graff M, Gigola G and Tanzola D (2020) Biological aspects of the life history of *Confluaria podicipina* (Cestoda, Hymenolepididae) from a hypersaline pampasic lagoon. *Pan-American Journal of Aquatic Sciences* 15, 54–63.

[ref26] Hartig F (2022) *Package DHARMa: residuals Diagnostics for Hierarchical (Multi-level/mixed) Regression Models*. Wien.

[ref27] Hudson PJ, Dobson AP and Lafferty KD (2006) Is a healthy ecosystem one that is rich in parasites? *Trends in Ecology and Evolution* 21, 381–385. 10.1016/j.tree.2006.04.007. 16713014

[ref28] Johnson A (1989) Movements of Greater flamingos (*Phoenicopterus ruber roseus*) in the western Palearctic. *Revue d’Ècologie* 44, 75–94.

[ref29] Koru E (2022) Cestode infection of the native brine shrimp (*Artemia parthenogenetica*) in Çamaltı Saltpan (İzmir/Türkiye). *Çanakkale Onsekiz Mart University Journal of Marine Sciences and Fisheries* 5, 56–66. 10.46384/jmsf.1084680.

[ref30] Levsen A, Cipriani P, Palomba M, Giulietti L, Storesund JE and Bao M (2022) Anisakid parasites (Nematoda: Anisakidae) in 3 commercially important gadid fish species from the southern Barents Sea, with emphasis on key infection drivers and spatial distribution within the hosts. *Parasitology* 149, 1942–1957. 10.1017/S0031182022001305. PMC10090642 36321524

[ref31] Lindén A and Mäntyniemi S (2011) Using the negative binomial distribution to model overdispersion in ecological count data. *Ecology* 92, 1414–1421. 10.1890/10-1831.1. 21870615

[ref32] Maksimova AP (1973) Branchiopod crustaceans as intermediate hosts of cestodes of the family Hymenolepididae. *Parazitologiya* 7, 349–353. 4272378

[ref33] Marcogliese DJ (2004) Parasites: Small players with crucial roles in the ecological theater. *EcoHealth* 1, 151–164. 10.1007/s10393-004-0028-3.

[ref34] Mastrolia A, Fiocca A, Pinna M and Basset A (2017) Salina dei Monaci e dune di Torre Colimena. *Transitional Waters Monographs* 3, 79–94.

[ref35] Morgulis A, Coulouris G, Raytselis Y, Madden TL, Agarwala R and Schäffer AA (2008) Database indexing for production MegaBLAST searches. *Bioinformatics* 24, 1757–1764. 10.1093/bioinformatics/btn322. PMC2696921 18567917

[ref36] Moscatello S and Belmonte G (2004) Active and resting stages of zooplankton and its seasonal evolution in a hypersaline temporary pond of the Mediterranean coast (the Vecchia Salina, SE Italy). *Scientia Marina* 68, 491–500. 10.3989/scimar.2004.68n4491.

[ref37] Moscatello S, Belmonte G and Mura G (2002) The co-occurrence of *Artemia parthenogenetica* and *Branchinella spinosa* (Branchiopoda: Anostraca) in a saline pond of south eastern Italy. *Hydrobiologia* 486, 201–206. 10.1023/A:1021307019891.

[ref38] Muñoz J, Gómez A, Green AJ, Figuerola J, Amat F and Rico C (2008) Phylogeography and local endemism of the native Mediterranean brine shrimp *Artemia salina* (Branchiopoda: Anostraca). *Molecular Ecology* 17, 3160–3177. 10.1111/j.1365-294X.2008.03818.x. 18510585

[ref39] Mura G (1995) Cestode parasitism (*Flamingolepis liguloides* Gervais, 1847 Spassky & Spasskaja 1954) in an *Artemia* population from south-western Sardinia. *International Journal of Salt Lake Research* 3, 191–200. 10.1007/BF01990494.

[ref40] Mura G, Baxevanis AD, Lopez GM, Hontoria F, Kappas I, Moscatello S, Fancello G, Amat F and Abatzopoulos TJ (2005) The use of a multidisciplinary approach for the characterization of a diploid parthenogenetic *Artemia* population from Torre Colimena (Apulia, Italy). *Journal of Plankton Research* 27, 895–907. 10.1093/plankt/fbi063.

[ref41] Mura G and Brecciaroli B (2004) Use of morphological characters for species separation within the genus Artemia (Crustacea, Branchiopoda). *Hydrobiologia* 520, 179–183.

[ref42] Nakagawa S and Schielzeth H (2013) A general and simple method for obtaining R2 from generalized linear mixed-effects models. *Methods in Ecology and Evolution* 4, 133–142. 10.1111/j.2041-210x.2012.00261.x.

[ref43] Nkouawa A, Haukisalmi V, Li T, Nakao M, Lavikainen A, Chen X, Henttonen H and Ito A (2016) Cryptic diversity in hymenolepidid tapeworms infecting humans. *Parasitology International* 65, 83–86. 10.1016/j.parint.2015.10.009. 26537836

[ref44] Palm HW and Rückert S (2009) A new approach to visualize ecosystem health by using parasites. *Parasitology Research* 105, 539–553. 10.1007/s00436-009-1423-z. 19337753

[ref45] Palm HW, Waeschenbach A, Olson PD and Littlewood DTJ (2009) Molecular phylogeny and evolution of the Trypanorhyncha Diesing, 1863 (Platyhelminthes: Cestoda). *Molecular Phylogenetics & Evolution* 52, 351–367. 10.1016/j.ympev.2009.01.019. 19489123

[ref46] Palomba M, Marchiori E, Tedesco P, Fioravanti M, Marcer F, Gustinelli A, Aco-Alburqueque R, Belli B, Canestrelli D, Santoro M, Cipriani P and Mattiucci S (2023) An update and ecological perspective on certain sentinel helminth endoparasites within the Mediterranean Sea. *Parasitology* 150, 1139–1157. 10.1017/S0031182023000951. PMC10941224 37942726

[ref47] R Core Team (2022) *R: A Language and Environment for Statistical Computing*. Vienna: R Foundation for Statistical Computing Austria.

[ref48] Redón S, Amat F, Hontoria F, Vasileva GP, Nikolov PN and Georgiev BB (2011) Participation of metanauplii and juvenile individuals of *Artemia parthenogenetica* (Branchiopoda) in the circulation of avian cestodes. *Parasitology Research* 108, 905–912. 10.1007/s00436-010-2132-3. 20981443

[ref49] Redón S, Berthelemy NJ, Mutafchiev Y, Amat F, Georgiev BB and Vasileva GP (2015) Helminth parasites of *Artemia franciscana* (Crustacea: Branchiopoda) in the Great Salt Lake, Utah: First data from the native range of this invader of European wetlands. *Folia Parasitologica* 62. 10.14411/fp.2015.030. 26040582

[ref50] Redón S, Quiroz M, Lukić D, Green AJ and Gajardo G (2024) Phylogenetic relationships of avian cestodes from brine shrimp and congruence with larval morphology. *Animals* 14, 397. 10.3390/ani14030397. PMC10854740 38338040

[ref51] Redón S, Vasileva GP, Georgiev BB and Gajardo G (2019) First report of cestode infection in the crustacean *Artemia persimilis* from Southern Chilean Patagonia and its relation with the Neotropical aquatic birds. *PeerJ* 7, e7395. 10.7717/peerj.7395. PMC6688596 31403003

[ref52] Robert F and Gabrion C (1991) Cestodoses de l’avifaune camarguaise. Rôle d’*Artemia* (Crustacea, Anostraca) et stratégies de rencontre hôte-parasite. *Annales de Parasitologie Humaine et Comparée* 66, 226–235. 10.1051/parasite/1991665226.

[ref53] Rode NO, Jabbour-Zahab R, Boyer L, Flaven É, Hontoria F, Van Stappen G, Dufresne F, Haag C and Lenormand T (2022) The origin of asexual brine shrimps. *The American Naturalist* 200, E52–E76. 10.1086/720268. 35905400

[ref54] Saccò M, White NE, Harrod C, Salazar G, Aguilar P, Cubillos CF, Meredith K, Baxter BK, Oren A, Anufriieva E, Shadrin N, Marambio-Alfaro Y, Bravo-Naranjo V and Allentoft ME (2021) Salt to conserve: A review on the ecology and preservation of hypersaline ecosystems. *Biological Reviews* 96, 2828–2850. 10.1111/brv.12780. 34747117

[ref55] Sánchez MI, Georgiev BB and Green AJ (2007) Avian cestodes affect the behaviour of their intermediate host Artemia parthenogenetica: An experimental study. *Behavioural Processes* 74, 293–299. 10.1016/j.beproc.2006.11.002. 17156940

[ref56] Sánchez MI, Green A and Castellanos E (2006) Temporal and spatial variation of an aquatic invertebrate community subjected to avian predation at the Odiel salt pans (SW Spain). *Archiv für Hydrobiologie* 166, 199–223. 10.1127/0003-9136/2006/0166-0199.

[ref57] Sánchez MI, Nikolov PN, Georgieva DD, Georgiev BB, Vasileva GP, Pankov P, Paracuellos M, Lafferty KD and Green AJ (2013) High prevalence of cestodes in *Artemia* spp. throughout the annual cycle: Relationship with abundance of avian final hosts. *Parasitology Research* 112, 1913–1923. 10.1007/s00436-013-3347-x. 23463137

[ref58] Sánchez MI, Paredes I, Lebouvier M and Green AJ (2016) Functional role of native and invasive filter-feeders, and the effect of parasites: Learning from hypersaline ecosystems. *PLOS ONE* 11, e0161478. 10.1371/journal.pone.0161478. PMC4999065 27560978

[ref59] Sánchez MI, Rode NO, Flaven E, Redón S, Amat F, Vasileva GP and Lenormand T (2012) Differential susceptibility to parasites of invasive and native species of *Artemia* living in sympatry: Consequences for the invasion of *A. franciscana* in the Mediterranean region. *Biological Invasions* 14, 1819–1829. 10.1007/s10530-012-0192-2.

[ref60] Sivakumar S, Hyland K and Schuster RK (2020) Tapeworm larvae in *Artemia franciscana* (Crustacea: Anostraca) in the Godolphin lakes of Dubai (United Arab Emirates) throughout an annual cycle. *Journal of Helminthology* 94, e5. 10.1017/S0022149X18000913. 30369336

[ref61] Spina F and Volponi S (2008) *Atlante della Migrazione degli Uccelli in Italia. 1. Non Passeriformi*. Rome: Ministero dell’Ambiente e della Tutela del Territorio e del Mare, Istituto Superiore per la Protezione e la Ricerca Ambientale (ISPRA).

[ref62] Sures B (2004) Environmental parasitology: Relevancy of parasites in monitoring environmental pollution. *Trends in Parasitology* 20, 170–177. 10.1016/j.pt.2004.01.014. 15099556

[ref63] Sures B (2008) Host-parasite interactions in polluted environments. *Journal of Fish Biology* 73, 2133–2142. 10.1111/j.1095-8649.2008.02057.x.

[ref64] Van der Auwera G, Chapelle S and De Wächter R (1994) Structure of the large ribosomal subunit RNA of *Phytophthora megasperma*, and phylogeny of the oomycetes. *FEBS Letters* 338, 133–136. 10.1016/0014-5793(94)80350-1. 8307170

[ref65] Young RT (1952) The larva of *Hymenolepis californicus* in the brine shrimp (*Artemia salina*). *Journal of the Washington Academy of Sciences* 42, 385–388.

[ref66] Zuur A, Ieno E and Smith G (2007) *Analysing Ecological Data*. Media, LLC, New York: Springer Science + Business.

